# Effects of climatic variables on weight loss: a global analysis

**DOI:** 10.1038/srep40708

**Published:** 2017-01-20

**Authors:** Morena Ustulin, Changwon Keum, Junghoon Woo, Jeong-taek Woo, Sang Youl Rhee

**Affiliations:** 1 Department of Medicine, Graduate School, Kyung Hee University, Seoul, Korea; 2 Division Biomedical Research Institute, Geference Inc., Seoul, Korea; 3 Data and Analytics, KPMG LLP, New York, New York, USA; 4 Department of Endocrinology and Metabolism, Kyung Hee University School of Medicine, Seoul, Korea

## Abstract

Several studies have analyzed the effects of weather on factors associated with weight loss. In this study, we directly analyzed the effect of weather on intentional weight loss using global-scale data provided by smartphone applications. Through Weather Underground API and the Noom Coach application, we extracted information on weather and body weight for each user located in each of several geographic areas on all login days. We identified meteorological information (pressure, precipitation, wind speed, dew point, and temperature) and self-monitored body weight data simultaneously. A linear mixed-effects model was performed analyzing 3274 subjects. Subjects in North America had higher initial BMIs than those of subjects in Eastern Asia. During the study period, most subjects who used the smartphone application experienced weight loss in a significant way (80.39%, p-value < 0.001). Subjects who infrequently recorded information about dinner had smaller variations than those of other subjects (β_freq.users dinner*time_ = 0.007, p-value < 0.001). Colder temperature, lower dew point, and higher values for wind speed and precipitation were significantly associated with weight loss. In conclusion, we found a direct and independent impact of meteorological conditions on intentional weight loss efforts on a global scale (not only on a local level).

The prevalence of obesity and overweight is increasing in developed and developing countries, leading to medical interventions, modifications of individual behaviours, and environmental changes^1^. Studies suggest that more than two-thirds of adults in the US are overweight or obese^2^. The rate of obesity among adults has risen from 13.4% in 1960 to 33.8% in 2010 in US. In UK, this rate passed from 15% in 1993 to 25.4% in 2010^3^. Obesity and overweight are associated with an increased incidence of cardiovascular diseases, type 2 diabetes mellitus, cancers (colon, endometrial, kidney), and other diseases. In US, obese adults had a higher risk of diabetes diagnosis than people with a normal weight (OR: 7.37) in 2001^4^.

In the literature, there are many studies that analyse as climatic factors can condition the lifestyle of people (such as physical activity)^5^ that affects the body weight. Nevertheless, these studies do not keep in consideration a direct effect among weather and changing of body weight. Also, these studies are limited to some particular areas without considering the differences that exits between geographical areas (different climatic conditions appear in the same season in several geographical areas, people located in distinct areas have different lifestyle, and so on). Therefore, in the literature, we find contrasting opinions. Having a sample on global scale and, in particular, considering the distinct areas in the analysis of association between weather and weight variation is a good way to have a general idea of the climatic effect on the weight loss. This knowledge could be useful to define a specific diet for people located in distinct zones subjected to different climatic conditions.

People tend to live a sedentary lifestyle and be conditioned by technology that around them. Developing health applications on the smartphones is a simple way to attract more people to take care own health in an easier way: for example people can control their calories intake continuously during the day, keep track of the physical activity and weight in every moment of the day providing therefore more detailed information. Also, the use of these applications can include also subjects that usually would not take care of their weight given the simplicity of these app.

Since it is not easy to keep track of the weather for each individual day, we used the data of the Weather Underground API and the technology of the smartphone to collect the information on people who were registered with a weight-loss application.

In this way, we could evaluate the effects of weather on weight loss using global-level information and overcome the limits of studies performed only in limited geographic areas.

## Methods

### Noom Coach

Noom Coach is one of the most popular applications for weight loss. Since 2012, it has been the top grossing health and fitness application in the Google Play Store, and more than 1 million people have installed it on their smartphones. This application has also been classified as the top weight loss app^6^,^7^.

During the first login, the user is asked to provide some information, including target body weight, current body weight, waist circumference, and daily food intake. Noom Coach is also able to record information on physical activity as the number of steps that the phone is carried daily. Using these data, the app gives a report on the user’s weight trend and a summary of diet with caloric intake for meals. It also gives some suggestions on physical activity that are useful to achieve target body weight.

Finally, this application is connected with a social network that allows users to find people and share their achievements.

### Study protocol

This study was performed to investigate the possible effects of climatic variables on intentional weight loss efforts using the Noom Coach application. Through this application, it has been possible to collect information regarding the locations of users (people who registered this application in their devices were aware that the anonymized information could be used in large-scale medical studies) and then to extract meteorological data from Weather Underground API for each login day^8^. Weather Underground API provides weather reports for cities around the world and allows users to access data and integrate them into other applications.

Users who installed the Noom Coach smartphone application from October 2012 to April 2014 were selected for this study. Users who used the application for at least 12 months, who provided their weights with information regarding meals at least once a month, and who were not 42 years of age (this is the default age given by the application if a value is not inputted) were included in this study.

The weather data included daily temperature, pressure, humidity, dew point, precipitation, wind speed, fog, and other variables. Not all weather variables were considered in this study since there were many missing data; users who had weather variables missing for at least one login day, after the linkage with the climatic information, were not considered in the analysis. For this reason, we chose the weather variables that allowed us to retain as many users as possible. Moreover, the variables that we used are those most commonly used in the literature^9^,^10^,^11^.

**Figure 1:**
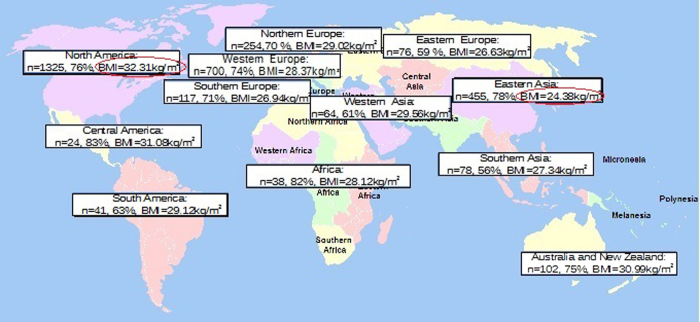
Geographic distribution of users. *United Nations geographical subregions (https://commons.wikimedia.org/wiki/File:United_Nations_geographical_subregions.png) is licensed under the Attribution-ShareAlike 3.0 Unported license. The license terms can be found on the following link: http://creativecommons.org/licenses/by-sa/3.0/”. **n = number of users, % female, initial BMI (kg/m^2^). ***The people located in different parts of Africa have been grouped into a single area since the sample size was small.

Using the location data, it was possible to divide users into several geographic areas [Fig. 1] following the United Nations Statistics Division (UNSD) based on the M49 coding classification (this study did not follow this geographic division completely, since there were not enough users in some areas).

Moreover, having both login days and latitude information, we divided the study period for each user (~1 year) into the usual seasons: summer, fall, winter, and spring. Since the seasons are not well defined close to the equator (latitude = 0), people with latitude values between −10 and 10 were excluded from this study. This choice was made after analyzing graphs in the literature that showed the variation in the Earth’s heat as a function of latitude during the year. Indeed, a review of the literature^12^ allowed us to choose the appropriate range of values empirically by elucidating how solar energy varies from tropical latitudes to polar latitudes during the year. Obviously, users located in the northern hemisphere had reversed seasons relative to those in the other hemisphere.

Through the application, we also extracted other user information: age, gender, calorie intake during meals (breakfast, lunch, and dinner), height, weight, and the login days considered in this study. Using the height and weight of each user, it was possible to compute the BMI on the first and final days of the follow-up period.

Through calorie intake, we classified individuals as frequent users if they regularly provided information about their meals. We defined a score for each day of use (score: 3 if the user logged information for all three meals, 2 if the user logged information for only two meals, 1 if the user logged information for only one meal, and 0 if all these data were missing), and we computed an average score for the entire follow-up period. Finally, we computed a median score to define a cutoff to obtain two groups containing similar numbers of people. A similar procedure was performed to define four other variables that take into account the input intake frequency for each meal and exercise information separately, classifying each user as frequent or not.

The latitude and longitude of the users’ locations should be available for mapping weather data. To remove outliers, we excluded users with values in the top and bottom 0.5% of all variables downloaded from the application and the top 1% of weight variances. Since it was important to keep track of the climatic variation during the follow-up period for each user, we decided to remove all users who had missing values for at least one of the following climatic variables: pressure, daily temperature, dew point, precipitation, or wind speed. In this way, we had complete information regarding weather for each user. People who never provided data for breakfast, lunch, or dinner were removed from the analysis, since it was not possible to obtain an estimate of the calorie intake for these individuals for each meal.

Using these criteria, we obtained 3274 users and 361,290 days [Supplementary Table].

### Statistical analysis

We performed an initial simple analysis to describe the general characteristics of the male and female users using Student’s t-test for the continuous variables (age, height, weight, and BMI) and the Chi-squared test for the categorical variables (BMI categories using the WHO BMC classification system). Then, we applied a paired t-test to compare the BMI values of the users at two time points (baseline and the end of follow-up) and to check for significant variations in BMI during the study.

Finally, we performed a mixed effects model to measure the possible effects of the weather variables (pressure, precipitation, wind speed, dew point, and temperature) on weight loss while also considering other variables: age, gender, geographic area, season, time of login, initial BMI, and average daily calories consumed at each meal. The season variable was considered a random effect, since users were located in different geographic areas, in which the seasonal effects are obviously different. For this reason, season was considered a source of variability.

In this model, we also evaluated the possible association between weight loss and the variable that characterized whether the user often provided information on meals during the day. In this way, it was possible to check whether regular use of the application had a positive effect on weight loss.

With this model, we could keep track of the BMI trend for each user considering the correlated measures for each subject, since we assumed that each user had his or her own pattern due to intrinsic individual characteristics. Using the coefficients of interaction between the time of login and the climatic predictors, it was possible to determine how BMI changed over time when the climatic variables varied. Since there were different time spaces between users (the number and days of login were heterogeneous in this study), the mixed effects model was the most appropriate method that we could use with this data structure. The entire analysis was performed using SAS software.

### Ethics Statement

This study was conducted in accordance with the guidelines laid down in the Declaration of Helsinki, the privacy policy of Noom Inc., and approved by the Kyung Hee University Hospital Institutional Review Board (KMC IRB 1435-04), which confirmed the absence of risk for the de-identified personal information leakage. The informed consents from the subjects were waived by the KMC IRB due to the retrospective design of this study

## Results

### Characteristics of the users

The users of the Noom Coach application were located in different geographic areas (with a total of 139 cities) [Fig. 1], primarily in North America (1325 people), Western Europe (700), and Eastern Asia (455).

People located in North America, Central America, Australia and New Zealand had frequently higher weights at baseline and were classified in “obesity class I” according to the WHO BMC classification system^13^. On the other hand, people who used the application in Eastern Asia had a lower initial BMI on average with respect to other users (BMI: 24.378; CI 95% = 24.030–24.727) and were classified as “normal users” [Supplementary Table].

A total of 3274 people (male: 854, female: 2420) were included in this study [Table 1]. The average number of logins for each user was 110 days, which means that each user had a login approximately every 3 days. Men used the application more often than did women (124 vs. 105 login days). As seen in Table 1, male users had an initial BMI higher than that of female users (p-value < 0.001); indeed, 33.18% of the women were classified in the “normal level” of the WHO BMC classification system, compared with only 11.71% of the men (p-value < 0.001). In addition, we noticed that at the end of the study this discrepancy between male and female’s weights was maintained (even if both achieved their goal) [Table 2].

During follow-up, we did not notice a difference regarding the use of the application between males and females when frequent and infrequent users were compared (frequent: people who regularly provided information on their meals). However, when we differentiated with respect to each meal, we noticed that male users tended to provide information regarding breakfast more often than female users did (p-value < 0.05).

Regarding calorie intake for all meals, we noticed a difference between female and male users (p-value < 0.001), with a higher amount of daily calories consumed by men than women, even if the calories counter embedded in the Noom application was not precise [Table 2].

### Effectiveness of the smartphone application for weight loss

People who used the smartphone application to lose body weight achieved their goal after approximately 1 year. The difference in BMIs measured at baseline and at the end of the follow-up period was significant (−2.079; p-value < 0.001; Table 2), and male users were more likely than female users to lose weight (85.60%). The same result was confirmed using a linear mixed-effects model [Table 3]. The “time” variable was associated significantly (p-value < 0.001) with BMI, showing a negative coefficient estimate (−0.015). Therefore, this negative association showed that the BMI decreased over time and subjects who used this app lost their weight.

### Effect of the weather and food intake self-tracking on weight loss

Using a linear mixed-effects model, it was possible to measure whether the variation in some climatic variables (pressure, temperature, dew point, precipitation, and wind speed) produces an effect on weight loss. From this model, we noticed that all of these weather variables were significant (p-value < 0.001) except for pressure [Table 3]. Using the coefficient of the time variable and the coefficients of interaction between climatic variables and time, we noticed that the variation in BMI, varying the time with a single unit, tended to be less negative when temperature increased. Therefore, low levels of temperature promoted weight loss. The same result was obtained with the dew point variable; the coefficient of interaction was positive, and therefore, low dew points supported weight loss.

In contrast, high values of precipitation and wind speed were associated with decreased BMI over time; the estimates of coefficients were negative (β_precipitation_ = −4*10^−5^; β_wind speed_ = −9.82*10^−7^).

**Figure 2:**
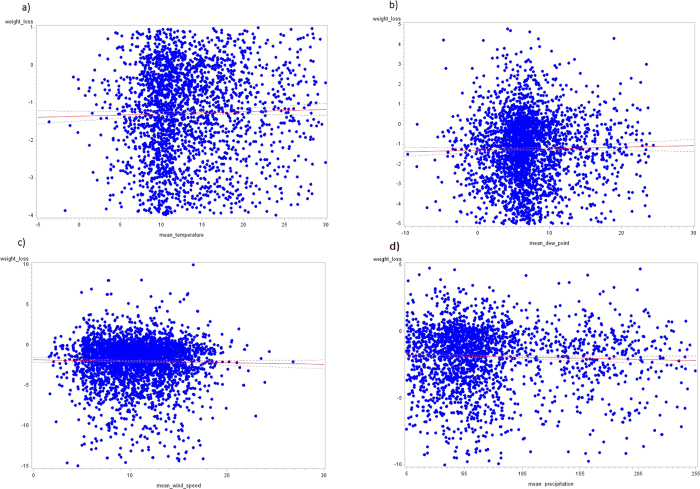
Scatter plots of weight loss and weather factors. *Weight loss: final BMI – initial BMI for each user, mean climatic value: average of the climatic values for each user and 95% CI (**a**) average temperature vs. weight loss, (**b**) average dew point vs. weight loss, (**c**) average wind speed vs. weight loss, (**d**) average precipitation vs. weight loss). The English in this document has been checked by at least two professional editors, both native speakers of English. For a certificate, please see: http://www.textcheck.com/certificate/SJ2UB2.

These results can be also observed in the graphs [Fig. 2], in which the values of weight loss (difference between final and initial BMI) for each subject and the mean values of the climatic variables are plotted: high values of temperature and dew point led to decreased weight loss, and high values for the other two climatic variables were associated with an increase in weight loss. In these scatterplots, we also noticed that most points for weight loss were less than zero, showing that many users lost weight.

Regarding the difference in BMI between frequent and infrequent users, we found that the BMIs of people who did not regularly provide information about each meal were higher than those of other users (β = 0.267; p < 0.001), and this relationship appeared at each login time (β = 5.6*10^−3^; p < 0.001). Moreover, we noticed that keeping track of information on dinner helped users lose weight: people who often provided data regarding dinner had lower BMIs than those who did not on each login day (β = 0.007; p < 0.001). These results were corrected by introducing other variables into the model such as initial BMI, area, season, average daily calories (for breakfast, lunch, and dinner), age, and gender.

## Discussion

Using the data from the app and the weather information, it was possible to keep track of the login days for each user and to obtain climatic data for each day of the study considering also the different seasons and location geographical areas of the subjects. The effect of climatic variables on BMI variation was analyzed: low values of temperature and dew point and high levels of precipitation and wind speed were associated with weight loss.

We also found that the use of this application helped people to lose weight, people showed a significant decrease of BMI at the end of study period. Our results agree with similar results obtained in another study^14^. One of the variables playing an important role in this analysis was the location of the user, which allowed us to keep track of the different diets, lifestyles, ethnicities and different climatic conditions of the distinct locations. In addition, the initial BMI was relevant to emphasize the negative trend of BMI over time. The amount of variation in BMI depended also on the initial weight: those who start a diet at a higher weight tend to have a higher variation in BMI compared with those with a lower initial weight.

The season can also affect BMI, and lifestyles change during different times of the year.

Unfortunately, we did not consider all users in this study: subjects who never provided information about their main meals (breakfast, lunch, and dinner) were discarded to avoid possible biases resulting from improper use of the app. In addition, the lack of information regarding climatic variables on some days led us to remove some users from the analysis, since it was important to have a complete trend for the weather variables to estimate possible associations between variations in the weather and BMI. Moreover, it was not easy to estimate missing data using the information at our disposal, since climatic variables have a high variability, particularly during the transition from one season to the next. Even though these choices led us to reduce the size of the sample, they allowed us to have maximal information for users during the follow-up period.

The model we used analyzed the trend for each user and computed an average effect of the evaluated associations, even if the follow-up time for individuals was different: users logged their information on the application during different times of the year (different baseline times) and with different frequencies.

The geographic area structure was useful to allow us to consider various diets and lifestyles that could influence BMI variation and in particular to consider that each area has different climatic conditions in the same season. Even if each user was located in the same place during the study, it was not guaranteed that ethnicity was defined by geographic area since the period of follow-up (around one year) was not very long, and the current migration rate is high.

Regarding the calories intake in the meals, the calories counter in the Noom application was not precise but anyway we considered this characteristic in the model since the variation of weight is conditioned by the total daily calories intake.

There are no studies in the literature measuring the direct effect of climatic variables on weight loss. Some studies attempted to analyze the possible association between weather conditions and physical activity (which is associated with body weight). For example, walking duration was higher during high temperature periods and longer during daylight hours in a sample of older people^15^. A study of urban teenagers in Baltimore showed that an increase in temperature led to more physical activities, whereas precipitation was negatively associated with physical activity^16^. Tucker *et al*.^5^ reviewed studies that analyzed this association and found that levels of physical activity varied with the season; notably, more physical activity was seen during summer. As reported in Tucker *et al*.^5^, the effect of the meteorological variables depended on the geographic area: Baranowski *et al*.^17^ showed an opposite association in Texas, where the average temperature is high in summer, since people avoid outdoor activities during this season. Ridgers *et al*.^18^ showed that the physical activity of children in Australia decreased in summer relative to winter, contrasting with previous studies. Some studies have analyzed the association between climatic variables and depression, a factor associated with obesity in de Wit *et al*.^19^. O’Hare *et al*.^20^ evaluated the effects of seasons and meteorological factors on depressive symptoms in older adults and found the highest levels of depression in the winter. The level of rainfall was also analyzed, and high levels of rain led to a greater number of depressive symptoms.

Therefore, the studies in the literature analysed only specific geographical areas founding contrasting results. They showed that each zone has distinct characteristics (such as different climatic conditions in the same season) that lead to get contrasting conclusions. Such contrasting conclusions were affected strongly by the fact of being in different geographical areas. Instead our study has been able to recruit subjects located in several zones providing a representative sample of the world. Also, we considered the “area” and “season” variables in our analysis. This allowed us to keep into account the differences between areas when we measured the association between weather and weight variation and avoid the possible biases due to the presence of different characteristics in each zone. In other words, our results are not affected by the fact of being in different areas because we corrected for such a possible bias.

## Additional Information

**How to cite this article**: Ustulin, M. *et al*. Effects of climatic variables on weight loss: a global analysis. *Sci. Rep.* **7**, 40708; doi: 10.1038/srep40708 (2017).

**Publisher's note:** Springer Nature remains neutral with regard to jurisdictional claims in published maps and institutional affiliations.

## Supplementary Material

Supplementary Information

## Figures and Tables

**Table 1:** Baseline characteristics of the participants in the study.

Characteristics	Male (n = 854)	Female (n = 2420)	p-value	Total (n = 3274)
Age (years) [95% Cl]	40.978 [40.242–41.714]	36.262 [35.805–36.719]	<0.001	37.492 [37.097–37.887]
Height (cm) [95% Cl]	177.70 [177.30–178.20]	164.70 [164.40–164.90]	<0.001	168.071 [167.767–168.375]
Weight (kg) [95% Cl]	96.518 [95.226–97.809]	79.096 [78.260–79.932]	<0.001	83.640 [82.889–84.391]
Baseline BMI (kg/m^2^)* [95% Cl]	30.476 [30.115–30.837]	29.122 [28.830–29.415]	<0.001	29.475 [29.239–29.712]
Underweight (BMI < 18.5)	0 (0%)	19 (0.78%)	0.009	19 (0.58%)
Normal (18.5 < BMI < 25)	100 (11.71%)	803 (33.18%)	<0.001	903 (27.58%)
Overweight (25 < BMI < 30)	372 (43.56%)	722 (29.83%)	<0.001	1094 (33.41%)
Obesity Class I (30 < BMI < 35)	237 (27.75%)	435 (17.98%)	<0.001	672 (20.53%)
Obesity Class II (35 < BMI < 40)	95 (11.12%)	228 (9.42%)	not significant	323 (9.87%)
Obesity Class III (BMI > 40)	50 (5.85%)	213 (8.80%)	0.007	263 (8.03%)

*BMI classification based on WHO criteria.

**Table 2:** Characteristics during and at the end of the follow-up period.

	Male (n = 854)	Female (n = 2420)	p-value	Total (n = 3274)
Breakfast calories (kcal/person insertion days) [95% Cl]	325.50 [317.0–334.0]	273.50 [269.30–277.60]	<0.001	287.02 [283.18–290.87]
Lunch calories (kcal/person insertion days) [95% Cl]	479.70 [469.70–489.70]	386.20 [381.40–391.10]	<0.001	410.62 [405.97–415.26]
Dinner calories (kcal/person insertion days) [95% Cl]	555.50 [543.50–567.50]	426.90 [421.20–432.60]	<0.001	460.43 [454.83–466.03]
Final BMI (kg/m^2^)* [95% Cl]	28.087 [27.756–28.417]	27.153 [26.889–27.417]	<0.001	27.397 [27.183–27.610]
Underweight (BMI < 18.5)	2 (0.23%)	49 (2.02%)	<0.001	51 (1.56%)
Normal (18.5 < BMI < 25)	239 (27.99%)	1065 (44.0%)	<0.001	1304 (39.83%)
Overweight (25 < BMI < 30)	385 (45.08%)	686 (28.34%)	<0.001	1071 (32.71%)
Obesity Class I (30 < BMI < 35)	151 (17.68%)	339 (14%)	0.009	490 (14.97%)
Obesity Class II (35 < BMI < 40)	52 (6.09%)	156 (6.45%)	not significant	208 (6.35%)
Obesity Class III (BMI > 40)	25 (2.93%)	124 (5.12%)	0.008	149 (4.55%)
**Paired T-test (difference between final and initial BMI)**				
	**Male (m = 854)**	**Female (n = 2420)**		**Total (n = 3274)**
Diff. BMI: end BMI-start BMI [CI 95%]	−2.389 [−2.588; −2.191](p < 0.001)	−1.969 [−2.084; −1.855] (p < 0.001)		−2.079 [−2.178; −1.979] (p < 0.001)
Users who lost weight (diff. BMI < 0)	731 (85.60%)	1901 (78.55%)		2632 (80.39%)
Users with stable weight (diff. BMI = 0)	8 (0.94%)	41 (1.69%)		49 (1.50%)
Users who gained weight (diff. BMI > 0)	115 (13.47%)	478 (19.75%)		593 (18.11%)

*Difference in the BMI of the subjects during the study period is significant (p < 0.001).

**Table 3:** Effects of weather on weight loss.

Linear mixed model using season as a random effect			
Variables*	Coefficients Estimate	CI 95%	p-value
Time	−0.015	−0.019; −0.009	<0.001
Temperature*time	5.7*10^−5^	4.7*10^−5^; 6.8*10^−5^	<0.001
Wind speed*time	−4*10^−5^	−0.4*10^−4^; −0.3*10^−4^	<0.001
Dew point*time	2.5*10^−5^	1.4*10^−5^; 3.5*10^−5^	<0.001
Precipitation*time	−9.82*10^−7^	**	<0.001
Frequent user (0 = no, 1 = yes), ref. = 1	0.267	0.124; 0.411	<0.001
Frequent user*time ref. = 1	5.6*10^−3^	0.005; 0.006	<0.001
Frequent user for dinner (0 = no, 1 = yes), ref. = 1	0.279	0.136; 0.422	<0.001
Frequent user for dinner*time ref. = 1	0.007	0.006; 0.007	<0.001

*Correcting for start BMI, area, season, average daily calories (for breakfast, lunch, and dinner), age, and gender. **Standard error is very close to zero.
