# Supplementary material for: Effects of climatic variables on weight loss: a global analysis
Source: Sci Rep. 2017 Jan 20;7:40708. doi: 10.1038/srep40708 (PMC5247768; doi:10.1038/srep40708)
Supplement: Supplementary Information [file srep40708-s1.pdf]

Distribution of initial and final BMIs for each location

Effects of climatic variables on weight loss: a global analysis

Morena Ustulin M.S., Changwon Keum M.S., Junghoon Woo, Ph.D., Jeong-taek Woo, M.D., Ph.D., Sang Youl Rhee, M.D., Ph.D.

Area	Tot. users	% F	Initial BMI (F, kg/m^2)	Initial BMI (M, kg/m^2)	Initial BMI (tot., kg/m^2)	Final BMI (F, kg/m^2)	Final BMI (M, kg/m^2)	Final BMI (tot, kg/m^2)
East Asia	455	78 %	23.56	27.26	24.38	22.37	25.62	23.09
West Asia	64	61 %	27.79	32.32	29.56	26.42	28.93	27.40
South Asia	78	56 %	26.78	28.08	27.34	25.72	26.49	26.05
North Europe	254	70 %	28.69	29.78	29.02	26.52	27.64	24.61
South Europe	117	71 %	25.64	30.09	26.94	24.27	27.98	25.35
West Europe	700	74 %	28.04	29.32	28.37	26.09	27.16	26.37
East Europe	76	59 %	25.35	28.49	26.63	23.65	25.99	24.61
Africa	38	82 %	27.53	30.73	28.12	26.35	28.22	26.69
Australia, New Zealand	102	75 %	30.53	32.44	30.99	27.99	29.57	28.38
South America	41	63 %	29.44	28.58	29.12	26.69	26.24	26.52
Central America	24	83 %	30.75	32.74	31.08	28.18	29.33	28.37
North America	1325	76 %	32.23	32.55	32.31	29.93	29.76	29.89
